# Permian scorpions from the Petrified Forest of Chemnitz, Germany

**DOI:** 10.1186/s12862-016-0634-z

**Published:** 2016-04-07

**Authors:** Jason A. Dunlop, David A. Legg, Paul A. Selden, Victor Fet, Joerg W. Schneider, Ronny Rößler

**Affiliations:** Museum für Naturkunde, Leibniz Institute for Research on Evolution and Biodiversity at the Humboldt University Berlin, Invalidenstrasse 43, D-10115 Berlin, Germany; Oxford University Museum of Natural History, Parks Road, Oxford, OX1 3PW UK; Paleontological Institute, University of Kansas, 1475 Jayhawk Boulevard, Lawrence, KS 66045 USA; Natural History Museum, Cromwell Road, London, SW7 5BD UK; Department of Biological Sciences, Marshall University, One John Marshall Drive, Huntington, WV 25755 USA; TU Bergakademie Freiberg, Geological Institute, Bernhard von Cotta-Straße, D-09599 Freiberg, Germany; Kazan Federal University, 420008 Kazan, Russia; Museum für Naturkunde, Moritzstraße 20, D-09111 Chemnitz, Germany

**Keywords:** Arachnida, Scorpiones, Burrows, Early Permian, Petrified Forest, Volcanism, Chemnitz, Germany

## Abstract

**Background:**

Paleozoic scorpions (Arachnida: Scorpiones) have been widely documented from the Carboniferous Period; which hosts a remarkable assemblage of more than sixty species including both putative stem- and crown-group fossils. By contrast the succeeding Permian Period is almost completely devoid of records, which are currently restricted to a trace fossil from the early Permian of New Mexico, USA and some limb fragments from the late Permian of the Vologda Region, Russia.

**Results:**

?*Opsieobuthus tungeri* sp. nov. from the Petrified Forest of Chemnitz, Germany represents the first complete body fossils of scorpions from the Permian. Explosive volcanism preserved these remarkable specimens in situ as part of the palaeosol horizon and bedrock of the Petrified Forest, immediately beneath the Zeisigwald tuff horizon. This dates to the early Permian (Sakmarian) or ca. 291 Ma. Intriguingly, the specimens were obtained from a palaeosol horizon with a compacted network of different-sized woody roots and thus have been preserved in situ in their likely life position, even within their original burrows. Differences in the structure of the comb-like pectines in the two fossils offer evidence for sexual dimorphism, and permit further inferences about the ecology and perhaps even the reproductive biology of these animals.

**Conclusions:**

As putative members of a Coal Measures genus, these fossils suggest that at least some Carboniferous scorpion lineages extended their range further into the Permian. This contributes towards a picture of scorpion evolution in which both basal and derived (orthostern) forms coexisted for quite some time; probably from the end of the Carboniferous through to at least the mid Triassic.

## Background

Scorpions are iconic arachnids with over two thousand living species distributed across eighteen different families [1]. They are also characterized by a fairly extensive fossil record, which extends back to at least the mid Silurian (ca. 430 Ma) [2] and renders them the oldest arachnids known to date. At present there are 131 valid species of fossil scorpion [3], although their distribution through time is far from homogeneous [4]. In particular, the Carboniferous Period (ca. 299–359 Ma) has yielded over sixty described species. By contrast, other geological periods have only around ten species or fewer. The Permian is particularly sparse with only two published records. From the early Permian (ca. 280 Ma) of Arizona there is an ichnospecies, *Alacranichnus braddyi* Lucas et al., 2013 [5], which the authors interpreted as the resting trace of a scorpion of uncertain taxonomic affinities. Late Permian (ca. 254–260 Ma) scorpion material from Isady in the Vologda Region of Russia [6] consists of limb fragments only and resembles specimens which were assigned to the Carboniferous genus *Eobuthus* Frič, 1904.

**Fig. 1:**
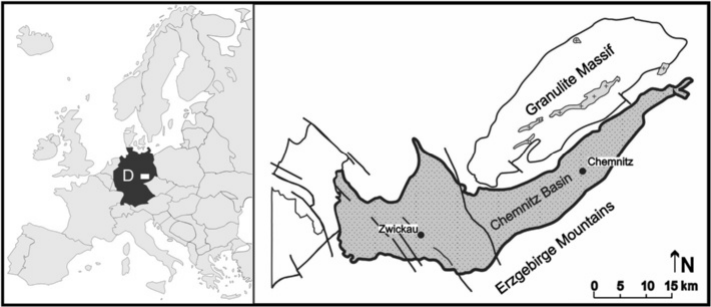
Location map of the fossil site in Chemnitz. The scorpions described here come from the Petrified Forest at Chemnitz-Hilbersdorf (Saxony), part of the Chemnitz Basin in eastern Germany

Here, we describe the first complete scorpion fossils from the Permian. Two well-preserved specimens, together with several fragments, were discovered within the early Permian (ca. 291 Ma) Leukersdorf Formation, the upper part of which contains the Petrified Forest of Chemnitz (Saxony, Germany). The scorpions come from a recently excavated (2008–11) locality in Chemnitz-Hilbersdorf, part of the wider Chemnitz Basin (Fig. 1), which represents an outstanding fossil assemblage buried almost instantaneously by pyroclastic flows. This remarkable autochthonous deposit preserves the most complete Permian forest ecosystem known to date. Fifty-three trunk bases, still standing upright in their places of growth and rooting in the underlying palaeosol, characterize this Fossil-Lagerstätte as a unique window into the past, offering insights into a lowland environment sheltering dense hygrophilous vegetation, as well as a diverse fauna of vertebrates, arthropods and gastropods. Several of the taxa found represent the first Permian record of their respective groups [7]. The Chemnitz fossils also occasionally preserve details of their putative habitat and life position, thus shedding light on their ecology and facilitating a reconstruction of this ancient forest ecosystem. Most fossil scorpions discovered hitherto were assumed to have been washed into their final depositional settings. We provide evidence that those from the Chemnitz Petrified Forest were buried in situ; perhaps even in their original burrows among the tree roots.

The present study is a continuation of on-going analyses of the Petrified Forest arthropods. As well as the scorpions described here, the fauna includes the youngest known trigonotarbid [8] – an extinct arachnid order – as well as whip scorpions (Thelyphonida), millipedes (Diplopoda), putative centipedes (Chilopoda) and remains of the giant myriapod *Arthropleura*. Study of these finds will not only enhance our understanding of a species-rich forested environment on mineral soils, but will shed light on synecological relationships within this volcanic-influenced landscape. This should contribute considerably to our view on food chains and evolutionary dynamics within the early Permian. Our scorpion fossils also have the potential to contribute to our understanding of the evolution of the group. As noted above, they are preceded by a species-rich Carboniferous fauna which includes many taxa belonging to early branching clades. However, a few Coal Measures scorpions evidently represent more derived taxa [3, 9, 10] sharing distinct apomorphies with living species. The Triassic yields the first, albeit controversial, record of a modern scorpion superfamily [11], while unequivocal representatives of modern families have been documented from the Cretaceous onwards (see Discussion). The implication is, therefore, of a shift from stem-group to crown-group scorpions going from the Paleozoic into the Mesozoic making the Permian a crucial time in scorpion evolution. A significant aim of the present study was to assess the position of these early Permian finds and to determine whether they are closer to the stem or the crown of the scorpion phylogenetic tree.

## Methods

### Material

Specimens were collected from an early Permian alluvial palaeosol immediately below the Zeisigwald tuff horizon, part of the Petrified Forest of Chemnitz, Saxony, Germany (50.85262 N, 12.94616 E). The two nearly complete scorpions are deposited in the Museum für Naturkunde Chemnitz under the repository numbers TA1116 and TA1126. They were informally named Jogi and Birgit, respectively, for the purposes of media attention. Part and counterpart exist for the complete specimens, revealing different diagnostic characters of the dorsal and ventral surfaces. Additionally, several scorpion fragments (e.g. TA1177, TA1187) probably represent imprints of exuvial remains and were spatially closely associated with specimen TA1116. Fossils were photographed using a Nikon D200 camera equipped with an AF-S Micro Nikkor 105 mm 1:2.8G lens and drawn using a Leica MZ12 stereomicroscope with a *camera lucida* attachment. Measurements are given in millimetres.

### Geological setting and preservation

**Fig. 2:**
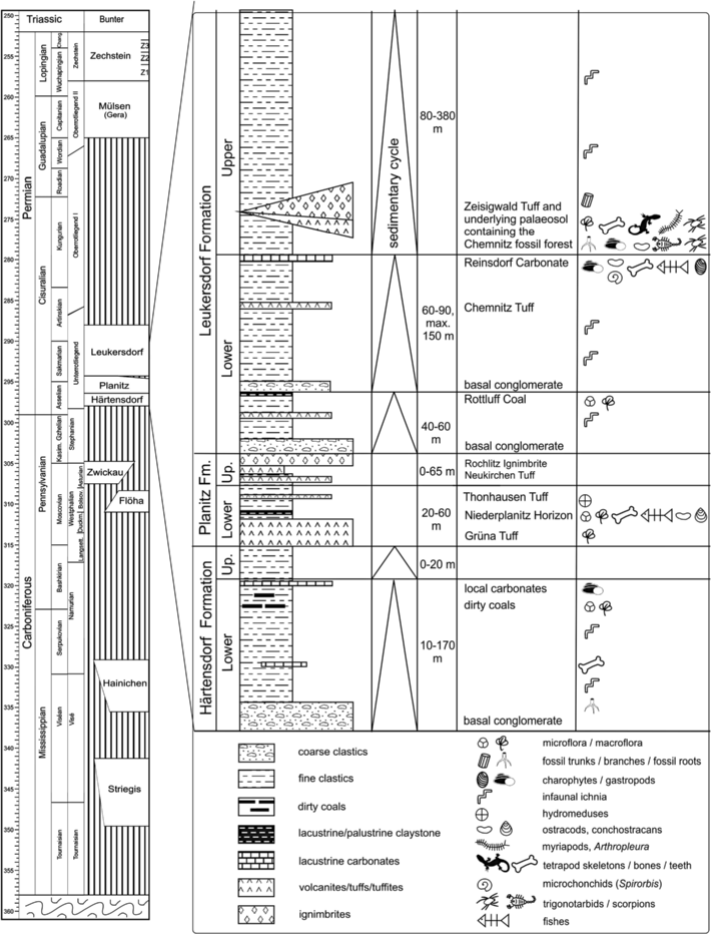
Stratigraphy of the Chemnitz site. The scorpions are part of the Petrified Forest ecosystem and are preserved immediately below the Zeisigwald Tuff Horizon; itself belonging to of the Leukersdorf Formation which is dated to the early Permian (Sakmarian), or ca. 291 Ma. Data adapted from [48]

Stratigraphically, the Petrified Forest of Chemnitz corresponds to the Sakmarian of the early Permian, because the entombing pyroclastics were isotopically dated at about 291.6 ± 1.8 Ma [12]. For further details of the geological background, stratigraphy and fossil record of this important Fossil-Lagerstätte see [7, 12–16]. The origin of the Chemnitz Petrified Forest is closely related to the explosive rhyolithic volcanism that occurred extensively during the early Permian [16, 17]. One of the eruptions in the area of present-day Chemnitz resulted in the formation of a pyroclastic flow sequence referred to as the Zeisigwald Tuff Horizon in the upper Leukersdorf Formation [18]. The Leukersdorf Formation (Fig. 2) consists of approximately 800 m of mainly sedimentary and subordinate volcanic deposits, dominated by red beds that were formed in a semiarid regional climate [18]. The alluvial plain sediments belong to the wet red beds type according to Schneider et al. [19] and were deposited during the middle Cisuralian wet phase D (*sensu* [16]). Apart from common tiny rootlets, this formation surprisingly rarely shows any basin-wide evidence of plant growth. This makes the Chemnitz Fossil-Lagerstätte an unusual, very local assemblage with a rich hygrophilous forest flora, a wet spot in the sense of DiMichele et al. [20].

**Fig. 3:**
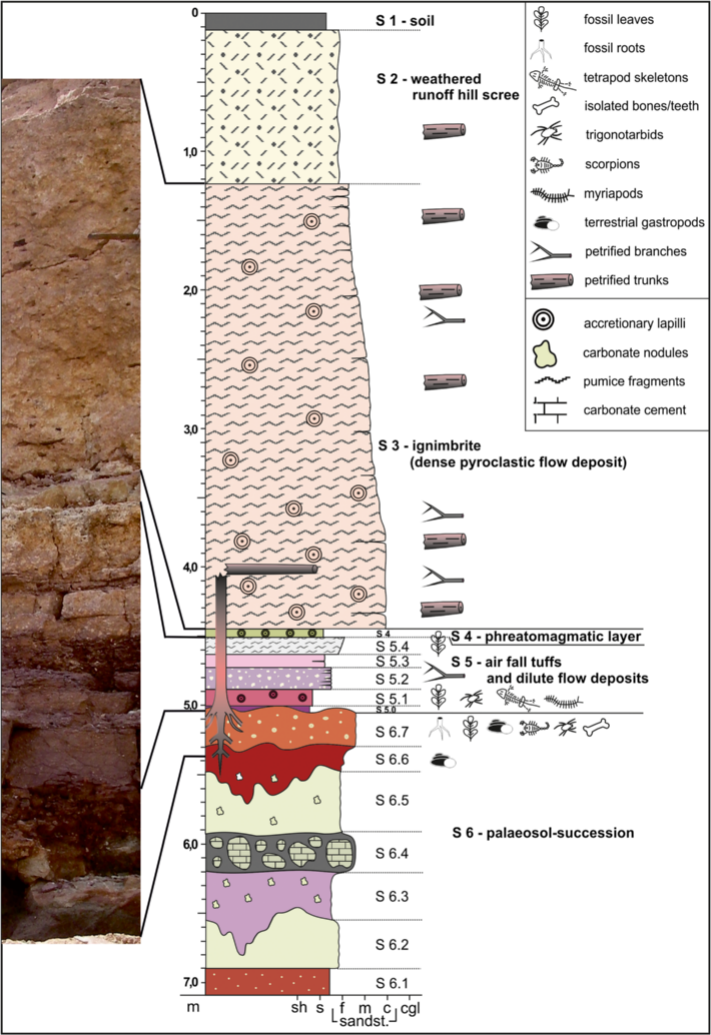
Geological section of the excavation. Detailed stratigraphic log of individual lithologies and biota at Chemnitz. Scorpions were found in the palaeosol horizon S 6.7, associated with tree roots in their original position. Higher levels in the sequence (S 3–5) reflect the explosive volcanism (e.g. air fall tuffs and pyroclastic flow deposits) believed to be responsible for preserving this remarkable biota in situ

The horizon from which our fossil scorpions were collected (Fig. 3: level S 6.7) represents the sedimentary basement of the Zeisigwald Tuff Horizon and therefore reflects the pre-eruptive environment [15]. This 1.85-m-thick palaeosol horizon consists of a varicoloured, fine, sandy siltstone with limited amounts of mudstone and fine sandstone. In some places, even matrix-supported quartz pebbles of centimetre size occur. The lower boundary to reddish-brown, well-bedded siltstones, the basin-wide typical wet red bed faces of the Leukersdorf Formation (Fig. 3: level S 6.1), is confluent. The whole palaeosol appears to be mainly without structure due to considerable bioturbation by various roots and soil organisms, such as gastropods. The sediment shows a light green to grey or purple mottling. Greenish leached parts are more intensely calcite cemented. A level with greenish grey, calcitic, millimetre-sized glaebules to decimetre-sized, internally micritic nodules with sparitic rootlet traces are intercalated 0.8 to 1 m below the top of this sedimentary unit (Fig. 3: level S 6.4). Top and base are gradational; nodules occasionally contain chert lenses of authigenic silica. The surface of this unit exhibits an undulating appearance.

**Fig. 4:**
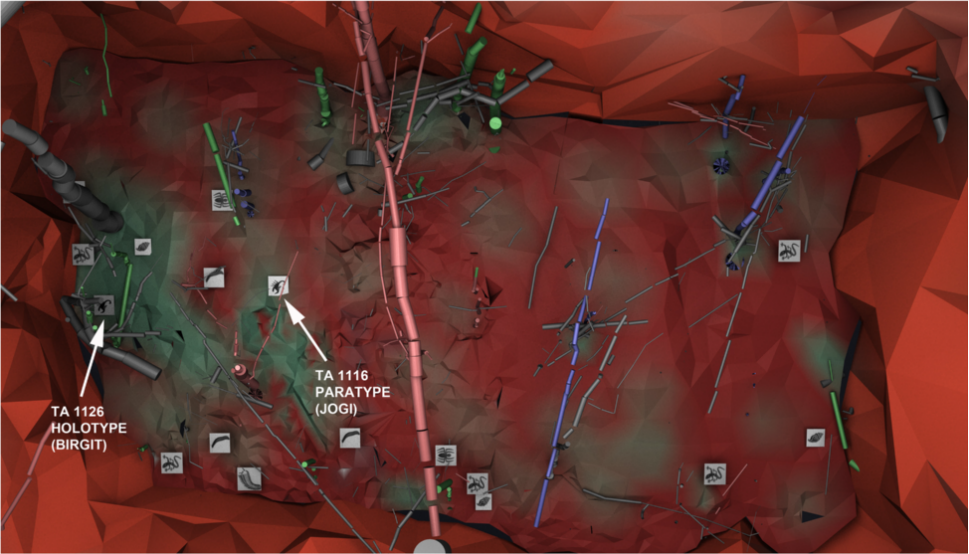
Three-dimensional plan view model from above of the excavation at the level of the palaeosol. Roots and trunks of the trees are shown interspersed with various animal fossils; all presumed to be close to their original life position. The original positions of the two complete scorpion fossils are arrowed, whereby the holotype was found beneath a gymnosperm complex root system in its original burrow. The scorpions, one trigonotarbid and the snails were all found at the same paleosol level. Image courtesy of Volker Annacker

The uppermost pale reddish brown 5 to 17 cm of the palaeosol are strongly lithified and represent the key horizon of our scorpion finds; the fossils were found a few centimetres beneath the sediment surface among compacted woody roots of cm to dm size. This horizon thus shows in situ root systems of several trunks (Fig. 4), such as cordaitalean and medullosan gymnosperms, psaroniaceous tree ferns and *Arthropitys*-type calamitaleans [15, 21]. The roots nearest to the trunks are found as petrifactions, but preservation commonly decreases in quality further away from the trunks so that only moulds, casts, or simply haloes remain. Close to the margin of petrified in situ trunks, the lithification of the sediment extends deeper. Distant from the trunks, compaction of all organic inclusions becomes more prominent. Plant litter rarely occurs as impressions without any organic matter. The supposed ground cover and leaf litter seem to be obscured due to a sub-Recent sliding surface between the palaeosol surface (Fig. 3: top of S 6.7) and the overlying pyroclastic rocks (Fig. 3: S 5.0 and S 5.1). Plant remains that could be identified were leafy conifer shoots of varying sizes, cordaitalean and pteridosperm leaf fragments, both cordaitalean and pteridosperm seeds, and small calamitalean leafy shoots [7]. Large, horizontal pieces of trunks in the upper part of the palaeosol may represent deadwood; surprisingly the anatomical preservation of these trunks improves the deeper they extend into the sediment, although they are significantly weathered or eroded at the boundary between the sediments and the pyroclastics. Disarticulated bones and toothed jaw fragments of vertebrates, plus casts and/or silicified or hematitic shell pseudomorphs of terrestrial gastropods are closely associated with root traces and deadwood trunks (Fig. 4). Another arachnid, probably a trigonotarbid *incertae sedis* preserved in a ventral position, completes the fossil record of the palaeosol horizon [15].

**Fig. 5:**
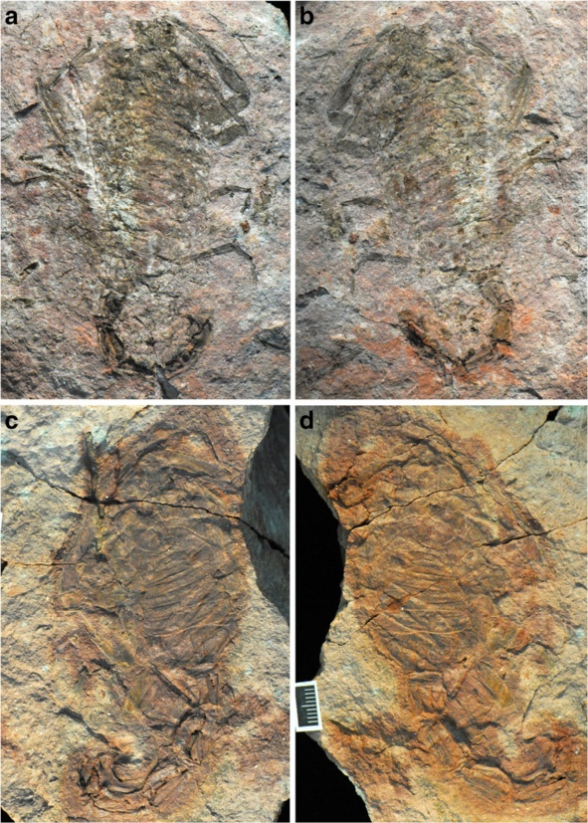
?*Opsieobuthus tungeri* sp. nov. **a**-**b**. Museum für Naturkunde Chemnitz, TA1126 (holotype). **c**-**d** TA 1116 (paratype). Both come from the palaeosol beneath the Zeisigwald Tuff Horizon, Leukersdorf Formation (lower Permian, Sakmarian) of the Chemnitz Petrified Forest, Saxony, Germany. The holo- and paratype were informally referred to as Birgit and Jogi respectively. Scale bar 10 mm

**Fig. 6:**
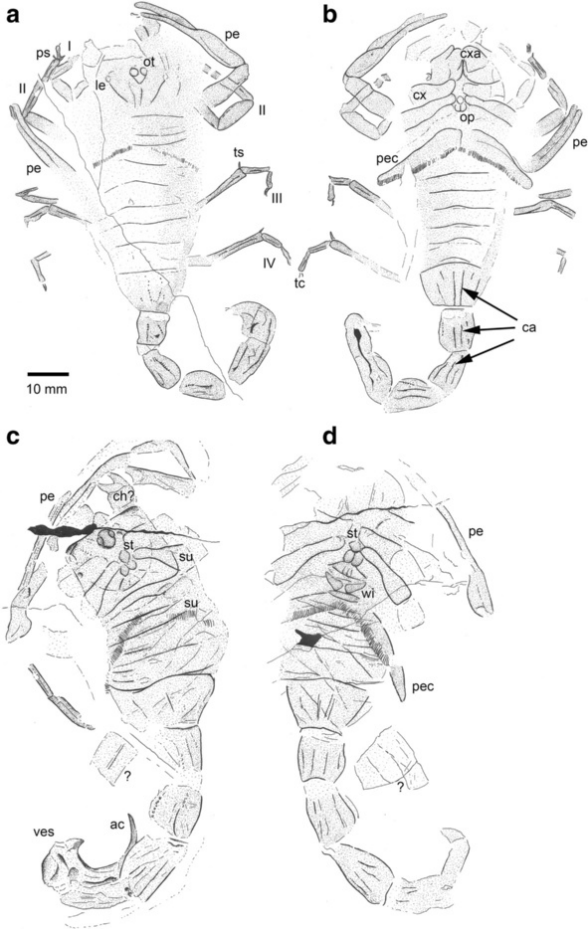
Interpretative drawings of the specimens shown in Fig. 5. **a**-**b** Museum für Naturkunde Chemnitz, TA1126 (holotype). **c**-**d** TA 1116 (paratype). Abbreviations: ac, aculeus; ca, carinae; ch ?, possible chelicera; cx, coxa; cxa, coxapophysis; le, lateral eyes; op, operculum; ot, ocular tubercle; pe, pedipalp; pec, pectines; ps, pedal spur; su, superimposed coxa and pectines; tc, tarsal claws; ts, tibial spur; ves, vesicle; wi, wing-like structure; ?, disarticulated cuticle fragment of uncertain affinity. Legs numbered from I–IV

The scorpion fossils (Figs. 5 and 6) are preserved as impressions without any cuticle remnants in a fine- to medium-grained siltstone. Since this largely bioturbated palaeosol substrate contains coarse-grained components, such as millimetre-sized mica crystals and rock fragments, the moulding of diagnostic surface features is inadequate in part. Although revealing certain three-dimensional aspects, dorsal and ventral morphologies are mainly superimposed.

## Results

### Systematic palaeontology

Order SCORPIONES Koch, 1850

Family CENTROMACHIDAE Petrunkevitch, 1953

Genus ?*Opsieobuthus* Kjellesvig-Waering [22]

### Remarks

The last major treatment of fossil scorpion systematics was the posthumous monograph of Kjellesvig-Waering [22] in which many new families, genera and species were proposed, often based on what seem to be trivial characters, possibly influenced by taphonomy and/or ontogeny. For example, the degree to which the ventral plates of the opisthosoma show lobation was a key feature of Kjellesvig-Waering’s scheme, but studies of modern scorpions [23] imply that this could just reflect differences between developmental instars in the fossils [24]. Furthermore, many of the Paleozoic scorpions in the literature are based on incomplete fossils, which may be restricted either to dorsal or ventral features only. Progress towards a cladistic classification has been made [25–27], but not all of these results were formally translated into synonymies and/or transfers, leaving authors to largely fall back on Kjellesvig-Waering’s monograph as the standard model.

A revision of Paleozoic scorpion phylogeny is in progress (Legg and Dunlop, in prep.; see also [24]) and we aim to place the new Chemnitz scorpions in the context of these provisional results. A difficulty we face is that while the fossils preserve the habitus quite well (Fig. 5), the coarseness of the preservation (see above) does not always recover fine details, such as the chelicerae or the more proximal limb articles, which would be useful for comparing the material to known fossil scorpion species. Despite these problems, we can identify some important features which rule out certain groups. For example, the large, anteriorly placed median eyes (Fig. 6a, c) exclude derived Carboniferous genera such as *Palaeopisthacanthus* Petrunkevitch, 1913 and *Compsoscorpius* Petrunkevitch, 1949 which have smaller eyes in a more posterior position [4, 22, 28]. Ventrally, the oral tube has strongly spatulate coxapophyses deriving from the first pair of leg coxae (Fig. 6b). The coxosternal region is quite well preserved – more so in the paratype (Figs. 5d and 6d) – and reveals a somewhat diamond-shaped sternum followed by paired, rounded genital opercula and a trapezoidal plate: the prepectinal plate *sensu* [22]. Most fossil (and living) scorpions have a triangular to pentagonal sternum. The diamond-shaped sternum in the new fossils is reminiscent of published descriptions of some *Eobuthus* species [22] and of a species assigned to *Opsieobuthus* Kjellesvig-Waering, 1986 which was itself originally described under *Eobuthus*.

*Eobuthus* may not be a valid genus [24]. Two of its three species, both from the Coal Measures of the Czech Republic, appear to be synonyms of scorpions belonging to other Bohemian genera, namely *Cyclophthalmus* Corda, 1835, which has a triangular sternum, and *Isobuthus* Frič, 1904 which has a pentagonal sternum. The third species, *Eobuthus holti* Pocock, 1911, from Lancashire in the UK, is an incomplete fossil in ventral view and is probably best treated as a *nomen dubium*. Note that the supposedly lost holotype was rediscovered [29], but adds little to the debate about its affinities. This leaves *Opsieobuthus pottsvillensis* (Moore, 1923) [30] from the late Carboniferous of the Clay City area of Indiana, USA as the closest match to our new material. Compared to published descriptions [22, 30] our fossil similarly reveals a diamond-shaped sternum followed by a putative bilobed genital operculum, a trapezoidal prepectinal plate partly divided along its posterior margin and additional wing-like structures at the front of the pectines (Fig. 6d); part of the pectinal plate *sensu* [22].

**Fig. 7:**
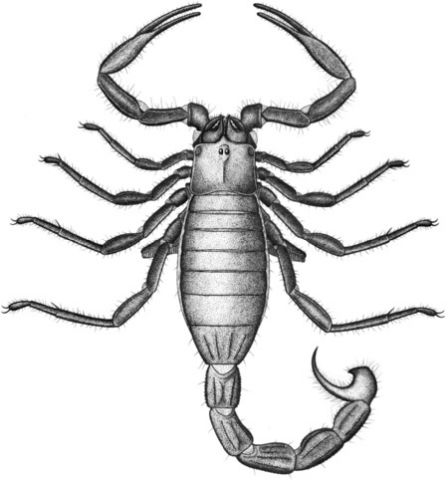
Appearance in life. Reconstruction of ?*Opsieobuthus tungeri* sp. nov. based on both the holo- and paratype, plus comparisons with other fossil and extant scorpions. Body length ca. 13 cm

However, we should note that there are alternative ways of interpreting these ventral structures ([26] Fig. 7). In the somewhat similar-looking *Centromachus euglyptus* (Peach, 1881) the bilobed structure was interpreted by Andrew Jeram as the posterior part of the sternum, the trapezoidal plate as the genital operculum and the paired structures behind this as the prepectinal plate. Resolving between these alternatives is beyond the scope of the present study, but is planned (Legg and Dunlop, in prep.) as part of a wider study of Paleozoic scorpions.

A further caveat to our assignment is that a putative diagnostic character of *Opsieobuthus* is the coxapophyses of the second pair of walking legs extending to the anterior tip of the first pair of spatulate walking leg coxapophyses [26]. In our new Chemnitz fossils, the second pair of coxapophyses only extends about half the length of the first apophyses (Fig. 6b). For this reason, we consider our generic assignment tentative, pending an assessment of the variability of this coxapophysis character within genera and thus its usefulness as a diagnostic taxonomic feature. Both the holotype and paratype are of a similar size and general habitus, and at least three shared characters imply that they are conspecific: (1) the long, slender pedipalps, (2) the very large pectines, and (3) the diamond-shaped sternum and the pair of sclerites immediately behind it.

?*Opsieobuthus tungeri* sp. nov.

LSID: urn:lsid:zoobank.org:act:48407D9C-E50B-4205-8C7E-236EF7259836

Rößler et al. [7] “Pulmonate scorpion” p. 831, fig. 12C.

2013 “Skorpion” Rößler, p. 6, fig. 5.

Luthardt et al. [15] “scorpion” p. 633, fig. 4C.

### Etymology

In honour of our friend the Chemnitz geologist Dipl. geol. Bernd Tunger, who worked extensively on the excavations of the Petrified Forest site and shared his huge experience in field geology for many years.

### Material

Museum für Naturkunde Chemnitz, Germany, nos. TA1126 (holotype: Birgit) and TA1116 (paratype: Jogi); leg. Volker Annacker and Ralph Kretzschmar. Additional material TA 1187 and TA 1177 (exuvial fragments).

### Horizon and locality

From the palaeosol beneath the Zeisigwald Tuff Horizon, Leukersdorf Formation, Petrified Forest of Chemnitz, Saxony, Germany. Early Permian (Sakmarian).

### Diagnosis

Species of ?*Opsieobuthus* with large, wing-like pectines extending laterally beyond the lateral margins of the body; each pectine probably with at least 50–60 individual pectinal teeth.

### Description

Holotype (TA 1126) an almost complete specimen of a moderately sized scorpion; total preserved length ca. 120 (inc. telson). Part and counterpart primarily preserve dorsal and ventral surfaces, respectively, but some features partly superimposed (Figs. 5a-b and 6a-b). Fossil appears to have become slightly disarticulated around prosoma–opisthosoma junction, such that visible number of tergites and sternites does not fit comfortably into the space available unless one were to invoke an additional segment, which should not be present in the scorpion body plan. Body length measurement should be treated as an estimate only. Most complete leg used for measurements of limb articles. Descriptions of ridges and/or tubercles are of the condition in life. Prosomal dorsal shield (carapace) subquadrate ca. 13 long; margins not well preserved. Anterior border, and the presence/absence of a median projection, equivocal. A number of transverse lines could represent the posterior margin of the carapace, or of one of the following tergites; thus, exact carapace dimensions unknown. Median ocular tubercle teardrop-shaped (Fig. 6a); length 4.2, width 3.8, tapering posteriorly and bearing large pair of median eye lenses; diameter of each lens 1.8. Narrow ridge on midline runs behind median eyes. Lateral eye tubercles possibly present as suboval features near lateral margins of dorsal shield (Fig. 6a), but precise number of lenses equivocal. Median ocular tubercle on raised, inverted subtriangular area of dorsal shield. Putative lateral eye tubercles positioned on anterolateral edges of same raised area. No other dorsal shield ornament preserved.

Coxae of pedipalp and leg I visible in outline. Coxa I with inflated, spatulate coxapophysis, flanked anterolaterally by pedipalp coxa. Coxa II with slender, pointed, mesally projecting coxapophysis; these pointed second coxapophyses thus lying between more expanded projections of first coxa, and extending about half way along their length (Fig. 6b). Leg coxae appear to increase in length from anterior to posterior, but distal margins difficult to trace. Sternum small, approximately diamond-shaped, length 1.9. Immediately behind sternum, bilobed genital operculum distinct, oval, slightly wider (2.6) than long (1.8). Genital operculum and pectines separated by gap (artifact?), hence any pectinal and/or prepectinal plate is equivocal. Pectines very large (Fig. 6b), wing-like, projecting beyond lateral margins of opisthosoma and superimposed onto dorsal surface of fossil. Length of each pectine ca. 22, maximum width proximally 6.0; pectines taper towards rounded distal end but no subdivision into marginal and/or median lamellae seen. Pectines highly dentate, each pectine with >50 narrow individual teeth; tooth lengths ca. 1.4.

Quadrate structure anterior to dorsal shield on right side may be chelicera. Pedipalpal chelae distinctly gracile (Fig. 6a). Manus slightly inflated, both fixed and movable fingers long and slender with slight inward curvature. Pedipalpal femur equivocal; patella subrectangular, length 12; length of tibia (manus + fixed finger) 27; length of tarsus (free finger) 14. Patella with hints of carinae near margins; manus of tibia with slight ridges close to lateral margins. Movable finger with row of tiny depressions on inner face, (setal sockets?). No trichobothrial pattern seen on pedipalp. Legs equally gracile (proximal articles poorly preserved). Preserved lengths of leg I articles: patella 6.4; tibia 0.8. Preserved lengths of leg II articles: patella 9.6; tibia 8.9; basitarsus 5.7; telotarsus 3.2. Leg II equivocal. Preserved lengths of leg III articles: tibia 10.7; basitarsus 6.1; telsotarsus 3.9. Preserved lengths of leg IV articles: tibia 13.3, basitarsus 5.0, tarsus 4.6. Patella of at least legs I and II inflated (as in modern scorpions), thickest at midlengths. Basitarsus of leg II terminates in pair of distal pedal spurs. Tibia and basitarsus of at least legs III and IV ornamented with longitudinal ridge. Tibia of legs III and IV expand slightly distally, also bearing short but prominent distal tibial spur at terminus. No spurs evident on basitarsus of these appendages. Basitasus of all legs (where preserved) slightly longer than telotarsus; latter terminating in pair of short, slender claws. Third claw (apotele) not evident.

Opisthosoma divided into meso- and metasoma. Mesosoma fairly slender; length ca. 43; maximum width ca. 20, but lateral margins poorly defined. Tergites and sternites largely without visible ornament; terminal mesosomal segment (i.e. segment 7) tapers posteriorly, dorsally and ventrally with four distinct carinae (Fig. 6a-b) formed from rows of raised denticles. Tergite boundaries not well resolved which, combined with superposition of ventral elements, makes precise length measurements difficult; tergites appear to lengthen posteriorly. Approximate lengths: first 4, second 4, third 5, fourth 4, fifth 4, sixth 6, seventh 9. Sternites with somewhat procurved posterior margins (approaching the protolobostern condition of Kjellesvig-Waering [22]) lengths ca. 4.3 to 5.7; seventh sternite almost 10. Sternites show no evidence of lung spiracles.

Metasoma (tail) with five segments: lengths 7.1, 8.9, 10, 11.3, and ca. 9, respectively; total preserved length ≥46, excluding telson. Fifth metasomal (preanal) segment not noticeably longer than preceding (fourth) segment (unlike in extant scorpions). Metasomal segments slightly wider posteriorly; each segment ornamented dorsally with distinct pair of carinae (Fig. 6a) formed from rows of raised denticles with two more carinae on lateral margins and four ventrally (Fig. 6b). Telson not well preserved, swollen vesicle region apparently tapers towards narrow aculeus (sting).

Paratype (TA 1116) almost complete specimen, total length ca. 130 (inc. telson). Part and counterpart mainly preserve dorsal and ventral surfaces, respectively; features superimposed (Figs. 5c-d and 6c-d). Paratype appears to have been foreshortened; mesosoma does not appear long enough to accommodate the assumed number of segments (seven). Again, body length measurement should be treated as an estimate only. Prosomal dorsal shield subquadrate, slightly wider than long, length 19.5, width 21.8, widening slightly posteriorly and anteriorly with triangular projection on midline (linguiform process *sensu* Kjellesvig-Waering [22]). Median ocular tubercle raised, positioned anteriorly, immediately behind anterior projection (Fig. 6c). Ocular tubercle teardrop-shaped; length 4.5, width 4.3, tapering posteriorly; bears pair of large median eye lenses, diameters 2.0. Oval areas towards anterolateral corners of dorsal shield could represent lateral eye tubercles, but details equivocal. Central area of dorsal shield slightly raised. Posterior margin of dorsal shield distinctly procurved. No other dorsal shield ornament preserved.

Coxae of pedipalps and of legs I–II equivocal. Coxae III and IV bell-shaped, widening distally; that of leg IV quite long, ca. 17. Sternum small (Fig. 6d), sub-diamond shaped, length ca 2.5. Immediately behind sternum, bilobed genital operculum, oval, slightly wider (4.1) than long (2.7). Prepectinal plate behind operculum, trapezoidal, length 2.7, maximum width ca. 9; medially divided on posterior margin. Pair of small, anterolaterally directed, triangular wing-like structures (pectinal plate?) anterior to pectines; length 4.5. Pectines very large (Fig. 6d), wing-like, projecting beyond lateral margins of opisthosoma and superimposed onto dorsal surface of fossil. Length of each pectine ca. 25, thus slightly longer but also thinner than in holotype; maximum width proximally 4.5. Pectines taper distally; no subdivision into marginal and/or median lamellae seen. Pectines highly dentate, each pectine bearing >40 narrow teeth (in life probably more present because teeth cannot be traced along whole pectine); tooth lengths ca. 2.2.

Structure on right side projecting from beneath prosomal dorsal shield interpreted as chelicera (Fig. 6c); robust, chelate, length ca. 10, maximum width ca. 6. Pedipalps gracile; manus slightly inflated, both fixed and movable fingers long, slender with slight inward curvature. Femur oblong, length ca. 16, maximum width 3.6; patella largely unknown; length of tibia (inc. fixed finger) ca. 40; length of movable finger (tarsus) ca. 25. Free finger with row of tiny denticles on inner surface. Most legs missing. Fragment of left leg (?) IV preserved; probably tibia (preserved length 12) and basitarsus (length 8.6).

Opisthosoma divided into meso- and metasoma. Mesosoma length ca. 32, but may be taphonomically foreshortened; width ca. 27; lateral margins poorly defined. Tergites and sternites largely without visible ornament, terminal mesosomal segment (segment 7) tapers posteriorly, at least ventrally with four distinct carinae. Tergites and sternites superimposed (thus difficult to resolve dorsal versus ventral elements and obtain precise length measurements); clear lengthening trend posteriorly. Approximate segment lengths: second 2.0, third 3.6, fourth 4.1, fifth 5.5, sixth 5.5, seventh 10.5. At least segment (?sternite) six with procurved posterior margin (approaching protolobostern condition of Kjellesvig-Waering [22]).

Metasoma (tail) with five segments: lengths 10.5, 14.5, 15.5 and 15.5, posteriormost not well preserved, apparently overlying telson; total metasoma length (exc. telson) ca. 64. Metasoma generally robust, segments slightly wider posteriorly. Where preserved, segments ornamented dorsally with pair of carinae formed by rows of raised denticles; four similar carinae ventrally. Telson very large (Fig. 6c), length ca. 25. Vesicle probably bulbous; aculeus long, curved, with prominent triangular subaculear tooth basally.

## Discussion

### Palaeoecology and environmental interpretation

**Fig. 8:**
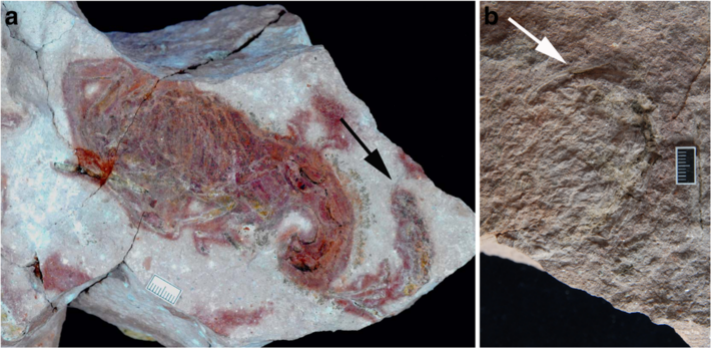
Evidence for ecdysis. **a** Alternative view of the paratype TA1116 under polarised light showing additional (?metasomal) cuticle fragments immediately behind the main body. **b** Additional specimen (TA1187) showing isolated remains of scorpion body segments; again perhaps from a moulted exuvium. Scale bars 10 mm

**Fig. 9:**
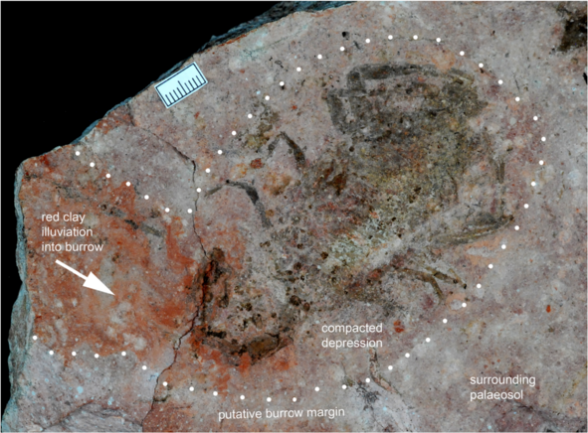
Evidence for being preserved in situ in a burrow. Alternative view of the holotype TA1126 showing its wider context in the matrix. The sediments surrounding the scorpion outline and define a putative burrow around this scorpion (delimitated by a white dotted line), which was discovered immediately below a woody gymnosperm root (cf. Fig. 4); whereby red-coloured clay appears to have been washed into the burrow entrance (arrow indicates direction of clay illuviation). Image taken under polarised light. Scale bar 10 mm

A reconstruction of the probable life appearance of ?*Opsieobuthus tungeri* sp. nov. is shown in Fig. 7. Applying the criteria of McCoy and Brandt [31] for recognizing fossil scorpion mortalities (cf. moulted exoskeletons) we can interpret the Chemnitz fossils as more likely to be mortalities. The pedipalps are preserved drawn in, rather than extended out, and the body is largely straight, without a curved mesosoma. We also have evidence for additional fragments of (?moulted) cuticle immediately behind TA 1116 (Fig. 8a) and the isolated accumulations of cuticle fragments in specimens such as TA1177 and TA1187 (Fig. 8b). At least TA1126, the holotype, was discovered in what was likely to have been its original life position within its natural habitat (Fig. 4). It was found in a compacted depression, positioned beneath a woody root of almost 6 cm width. This depression is located 2 to 6 cm below the flattened root and about 8 cm beneath the palaeosol surface. From the entrance to the distal range of this putative burrow there is recognizably a gradually decreasing amount of red-coloured clay (clearly visible under polarised light; see Fig. 9). Because this kind of a pure clay layer has not been observed anywhere in the soil horizon, it is interpreted as clay illuviation into the (compacted) scorpion burrow. In support of this illuviation interpretation there is also a difference in grain size between the burrow infill – the red clay layer – and the surrounding palaeosol.

The width of this clay layer corresponds exactly to the body width of the scorpion TA1126 which supports our interpretation of the animal being buried in its burrow. The putative burrow extends for several cm behind the scorpion body while maintaining this same width, i.e. it does not form a halo around the body as would be expected if this effect was caused by decay. Furthermore, we know of no decay-related (bio)chemical reaction which would yield illicit clay; while at the same time it is common knowledge that hollows in soils caused by burrowing organism, or by decayed roots or other plant remains, are framed (or more or less completely filled) by clay illuviations as in our material. Modern burrowing scorpions often moult inside their burrows [32]. The additional presence of isolated body remains (also preserved as imprints and here interpreted as further possible moulted exuvia remains) inside the burrow range also points to the in situ situation of TA1126.

In relation to the preservation of the scorpions, the volcanic ash fall effectively acted as a ‘coffin lid’ to cover the material. The palaeosol itself was too coarse-grained to reveal fine anatomical details, and at the same time was apparently too aerated/oxidised to preserve any organic polymers from the original cuticle. According to the general litho- and biofacies characteristics of the sedimentary unit containing the horizon yielding the scorpions, this layer is interpreted as an alluvial palaeosol [15]. The most conspicuous feature is the common presence of roots in different forms of preservation, intensive colour mottling, and the occurrence of carbonate glaebules of different sizes. The rooting of plants and other processes involved in soil formation, such as swelling, shrinkage, pedoturbation or various animal activity, have altered or completely destroyed most pre-existing sedimentary structures. Both the red and purple mottling of the muddy sediment and the loss of organic matter indicate periods of soil oxidation that are usually observed in well-drained surface soils. The lack of carbonaceous root preservation and rubefaction on one hand, and evidence of periods of more sustained plant growth, waterlogging, and the lack of visible endogenous ichnia of the *Scoyenia*-ichnofacies on the other, point to a polygenetic palaeosol that formed at a relatively low accommodation rate. The last phase of palaeosol formation took place during seasonally high groundwater levels [15].

Eventually, this palaeosol supported a dense vegetation dominated by hygrophilous elements, but did not develop any organic deposits such as peat, pointing to a nearly complete recycling rate of the plant litter within this forested habitat. As remnants of the primary sediment composition and structures in both the soil horizon and the sediments beneath the palaeosol indicate, soil formation and growth of the forest took place in a special local sedimentary environment of the typical Leukersdorf Formation wet red beds. Deposition was dominated by suspension, in places also with a minor bedload of sandy-pebbly braided river channels, and caused a multistacked, fine-grained deposit to form in a distal floodplain environment. Recent investigations based on specific geochemical proxies and anatomical characteristics of the plant fossils provided evidence of strong seasonality of this environment and revealed a mean annual precipitation of 800 to 1100 mm [15].

### Terrestrial predatory arachnids

The mode of preservation and depositional environment documented here in the Petrified Forest of Chemnitz contrasts markedly with the environment interpreted for the majority of the Paleozoic scorpions. There remains a long dispute regarding the original habitats of Paleozoic scorpions with some authors, especially Kjellesvig-Waering [22], interpreting almost all Paleozoic scorpions as aquatic. By contrast, comparative anatomy [33] has been used to argue that the arachnid book-lung has a single origin, which would imply that all scorpions were terrestrial throughout their geological history. Anatomical characters which could support a marine/aquatic or terrestrial mode of life have been proposed [34]. However, recently, even taxa whose mode of life appeared to be well substantiated have come under discussion again. For example, the Devonian *Palaeoscorpius devonicus* Lehmann, 1944 was, for many years, regarded as the epitome of a marine form, in part based on the presence of unequivocally marine animals in the Hunsrück palaeoenvironment, but restudy has challenged this interpretation by finding evidence for possible lungs in this scorpion [35]. At the same time, another Devonian genus, *Waeringoscorpio* Størmer, 1970 bizarrely seems to show features consistent with having external gills similar to those of certain modern insects which have become secondarily adapted for benthic aquatic life [36]. It is possible that scorpions occupied a wider range of habitats in the past than they do today. Even clearly terrestrial forms, such as those from the Viséan of Scotland [26], or representatives of modern families from the Lower Cretaceous Crato Formation of Brazil [37], were obviously washed into their lacustrine depositional areas alongside the many associated plant remains.

**Fig. 10:**
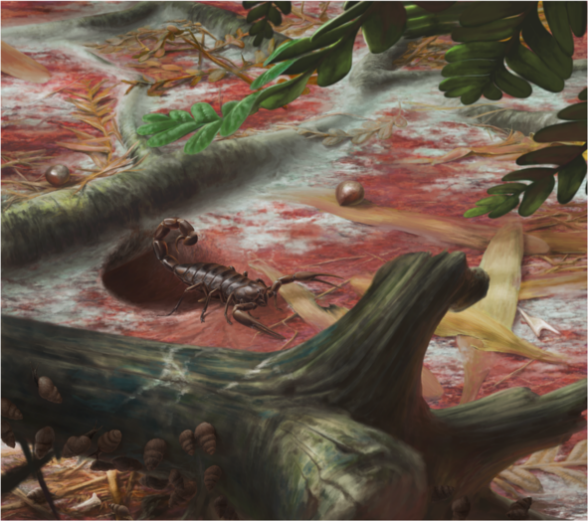
Ecological reconstruction. ?*Opsieobuthus tungeri* sp. nov. placed in its suggested original environment at the mouth of a burrow on the forest floor. Drawing by Frederik Spindler

The Chemnitz scorpions come from an unequivocally terrestrial palaeoenvironment, significantly without any indication of large standing water bodies, but with multiple lines of evidence (calcic to ferric palaeosols, frequent growth rings in woody plants) for at least seasonally dry conditions. A further reconstruction of ?*Opsieobuthus tungeri* sp. nov. in its original environmental setting is shown in Fig. 10. Scorpions today inhabit a wide range of environments from hot, dry deserts to warm, humid rainforests. Extant scorpions commonly live in burrows (see e.g. [38] and references therein), either excavating them themselves or using existing burrows made by other animals. They may spend the best parts of their lives within these retreats, emerging only infrequently and often at night in order to hunt [39]. Burrowing scorpions of around 12 cm body length (or larger) are very common in the tropics, such as *Heterometrus* spp. in Asia (Scorpionidae: burrows of up to 40 cm in wet soils), *Pandinus* spp. in Africa (Scorpionidae), or *Brotheas* spp. in South America (Chactidae). They all make burrows in the organic soil, fine mineral soil, and inside rotten logs. Presence of exuvia (moult skin) next to one fossil specimen at Chemnitz (Fig. 8a) suggests an ecdysis hideout during moulting.

Scorpions today are invariably non-specialized predators. Modern species mostly feed on other arthropods, but will also take gastropods and even small vertebrates. Among fossil animals in the Chemnitz Fossil-Lagerstätte, these large (~12 cm) scorpions were probably among the top invertebrate predators. Their prey could include smaller animals: insects (fossils not found so far), myriapods and gastropods. We do not know if they were predated upon (in comparable modern habitats, large scorpions are often the top predators) but different vertebrates are also known from this fauna. Scorpion density at Chemnitz can be formally estimated as not less than one per 200 m^2^ (i.e. at least two specimens in a *c*. 400 m^2^ plot: Fig. 4). Such a range is not uncommon in modern forests. Among extant scorpions, densities can be higher (deserts >1 m^2^; littoral *c*. 10 m^2^), i.e. they are most common predators in these environments.

### Morphological adaptations and dimorphism

**Fig. 11:**
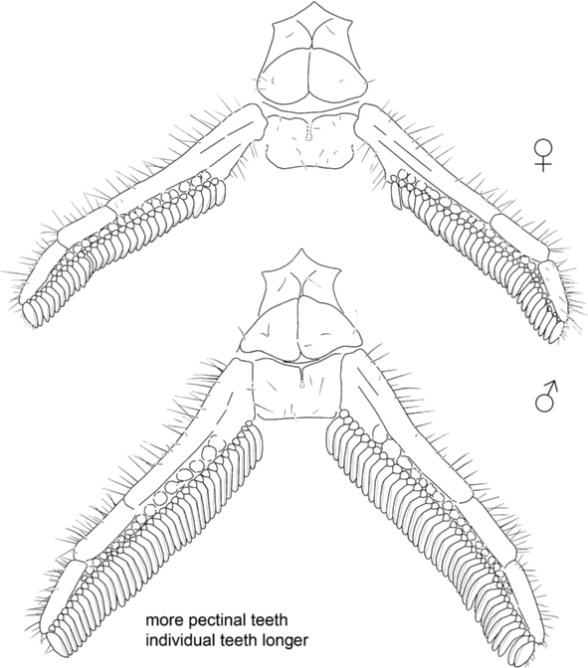
Pectines of living scorpions. *Hadrurus anzaborrego* Soleglad et al., 2011 [49] (Scorpiones: Caraboctonidae) from California, USA; above, the female, below, the male. Like the paratype of the new fossil species, the male here has more pectinal teeth and the individual teeth are slightly longer. After ([49]: figs. 45–46; courtesy of Michael E. Soleglad)

Pectines are unique to scorpions and commonly preserved in Carboniferous forms. Their presence may indicate a terrestrial (litter) adaptation for prey/mate localization and general olfactory orientation. The number of pectinal plates (teeth) on these organs is generally species-specific; with slight variation and some indication the number increases slightly with age. Furthermore, the number of pectinal teeth in extant scorpions is usually sexually dimorphic (Fig. 11); males always have a higher number of teeth, which also are markedly longer than those of females. A larger size (length) of male pectinal teeth allows them to house a considerably higher number of chemosensory sensilla within much larger sensory fields. Although only two complete specimens are known from Chemnitz, their similar size and similar habitus suggest that these fossils are probably conspecific (see also above). Nevertheless, there are a few subtle differences between the holo- and paratype and, in the light of what is known about modern scorpion pectines, it is interesting to speculate whether we have a male and a female, expressing sexually dimorphic characters. In particular the pectines of the paratype appear to be longer and thinner than those of the holotype (Fig. 6b), and to express the male character (see above) of slightly longer pectinal teeth (Fig. 6d). If this interpretation is correct, with a female holotype and male paratype, then the informal names Birgit and Jogi were assigned to the correct genders.

In a wider context, the number of pectinal teeth appears to be very high (50–70 per comb [21]) in at least some of the extinct mesoscorpions; a group which can be defined [26] on anteriorly located median eyes, 30–60 ocelli in each lateral-eye group (when present), and a preanal segment which is equal to or shorter than, the preceeding postabdominal segment. Our new material supports this basic assertion in documenting ca. 40–50 teeth borne on noticeably large pectines (see Description). The number of teeth in Orthosterni of comparable size is lower; maximal pectinal teeth numbers in extant males reach over 40. The exceptional, highest number of 45–58, in extant scorpions has been recorded in males of the South American species *Brachistosternus multidentatus* Maury, 1984 (Bothriuridae) [40].

The presence of two, probably conspecific, specimens assignable to different sexes implies a resident, reproducing population. They were found within ca. 2 m from one another (Fig. 4) and may thus even be a mating couple; many extant scorpions, especially of this large size, would normally maintain a larger distance from one another (R. Teruel, pers. comm.). Their preservation in situ and in close proximity – one male and another female, the female perhaps even being freshly moulted if the cuticle fragments are not so old and originate from the same animal – could even be the earliest tentative evidence of mate-guarding behaviour, a phenomenon first reported in scorpions by Benton [41]. Mate-guarding involves an adult male detecting a subadult female and staying with her in the same shelter to prevent other males from entering until she moults to maturity. Rolando Teruel (pers. comm.) observed mate-guarding (always by the male) in essentially all Caribbean species of Buthidae and Scorpionidae.

### Scorpion evolution

A major unresolved question for fossil scorpion research is whether the observed peak of Carboniferous diversity reflects a genuine period of scorpion diversity, or whether it is an artifact of sampling bias resulting from coal mining and the huge mine dumps and/or unreliable taxonomy. In favour of the latter hypothesis is the fact that many fossil species stem from the work of Kjellesvig-Waering [22] and Petrunkevitch [28] on the Mississippian of Scotland and the late Carboniferous Coal Measures of Europe and North America in particular. Both authors tended to recognise multiple species, often diagnosed on dubious characters. Revision has invariably reduced the observed diversity, such as in the case study of *Compsoscorpius buthiformis* (Pocock, 1911) from the British Middle Coal Measures for which nine junior synonyms could be recognised [4].

At the same time, the Carboniferous does appear to have been a crucial time of transition for these animals. As noted above, Kjellesvig-Waering [22] regarded almost all Paleozoic scorpions as aquatic and developed a typological scheme of families and superfamilies. Translating these, commonly monotypic, groups into a meaningful phylogenetic classification is still a work in progress. Three major lineages of fossil scorpions were recognised by Jeram [26] termed palaeo-, meso- and neoscorpions, and defined by accumulations of apomorphic characters to yield increasingly modern-looking species. A modification of these groups, recognising a series of increasingly derived clades at a finer level of resolution, can be found in the thesis of Legg [24]. This (unpublished) model includes higher taxa successively defined by the presence of two pairs of coxapophyses, the presence of a pentagonal sternum, and finally the crown-group orthosterns which are defined by book lungs opening in the middle of the sternites. Fossils assignable to of all of these grades can be found coexisting during the late Carboniferous. Thus the elevated number of fossil scorpion species could reflect a genuinely wider range of scorpion body plans at this time. Essentially, current data suggests that both stem- and crown-group species lived alongside one another in the Coal Measures. With this in mind, our new Permian finds are of particular interest in revealing whether one of the more basal scorpion lineages outlasted the Coal Measures, or whether the decline of the coal forests witnessed – or perhaps even induced – radiations of scorpions much closer to the living clades.

Our results suggest that the Chemnitz scorpions are probably best placed in a genus previously known from the Coal Measures: *Opsieobuthus* (see Systematics). In other words, these new Permian fossils express a mixture of plesiomorphic and derived characters including a number of typical features for Coal Measures scorpions, such as large and anteriorly positioned median eyes, spatulate coxapophyses on the first pair of legs, and the absence of an elongate preanal segment prior to the telson. Unfortunately, the position of the book lung spiracles (either marginal on the sternites or within the sternites, as in modern (orthostern) species is not resolvable in the Chemnitz fossils. Nevertheless, the implication is that at least one stem-group lineage did outlast the Carboniferous coal swamps, and was still present in the early Permian (ca. 291 Ma) of Germany.

This condition is also reflected in the Triassic *Mesophonus* Wills, 1910 [42] scorpions from the Lower Keuper (ca. 230 Ma) of England [42, 43], and the slightly older (Anisian: ca. 247 Ma) *Gallioscorpio voltzi* Lourenço and Gall, 2004 [11] from the Buntsandstein of the Vosges in France. These fossils, both mesoscorpions in traditional terminology, also retain evidently plesiomorphic character states, such as anteriorly placed median eyes and compound lateral eyes. In this context, one could argue that stem-group scorpions maintained a presence until at least the middle part of the Triassic period. At the same time, the early Triassic also yields the oldest putative member of a modern scorpion superfamily: *Protobuthus elegans* Lourenço and Gall, 2004 [11], also from the Vosges. However, the exact position of this fossil, assigned to an extinct family, Protobuthidae within the superfamily Buthoidea, has not been tested cladistically and its buthoid affinities have been questioned [44]. Jurassic records of scorpions are extremely sparse and have either been shown to be misidentifications, or are poorly preserved and not referable to any particular higher taxon [45]. Demonstrably modern scorpion families first appear as Chactidae and Hemiscorpiidae from the ca. 115 Ma Early Cretaceous Crato Formation of Brazil [37], with Chaerilidae reported from the ca. 99 Ma Late Cretaceous Burmese amber from Myanmar [46]. Note that Burmese amber also yields a number of putatively extinct family groups (reviewed by [47]). Modern families such as Buthidae begin to appear in subsequent Cenozoic deposits like the Baltic and Dominican Republic amber.

## Conclusions

The Permian Petrified Forest of Chemnitz hosted moderately large (ca. 12 cm long) scorpions which were probably notable predators on other arthropods, and perhaps even other small invertebrates and vertebrates within this ca. 291 Ma ecosystem. The fossils were preserved through explosive volcanism and there is evidence that they may even lie within their original burrows located under extensive networks of tree roots. Burrowing behaviour fits well with modern scorpion ecology. Notable are the very large pectines, which can be used to diagnose these fossils as a new species, probably belonging to the genus *Opsieobuthus*. Details of the pectinal teeth imply sexual dimorphism, with both a putative male and a female preserved in situ in close proximity to one another and thus conceivably even representing a mating pair. In a wider context, these significant finds represent the first complete fossil scorpions from the Permian and suggest that at least one Carboniferous genus extended its range beyond the time of the traditional Euramerican coal forests. This, in turn, contributes towards an emerging picture of a long (at least 80 million year?) overlap between the more basal scorpions, characterised by features such as anteriorly positioned median eyes, compound lateral eyes, and the absence of a long preanal segment, which lasted well into the Triassic, and the anatomically modern (i.e. orthostern) scorpion clade which first appeared towards the end of the Carboniferous.
